# PROTOCOL: Systematic review of methods to reduce risk of bias in knowledge translation interventional studies in health‐related issues

**DOI:** 10.1002/cl2.1236

**Published:** 2022-04-12

**Authors:** Ayat Ahmadi, Bahareh Yazdizadeh, Leila Doshmangir, Reza Majdzadeh, Shabnam Asghari

**Affiliations:** ^1^ Knowledge Utilization Research Center Tehran University of Medical Sciences Tehran Iran; ^2^ Department of Health Policy & Management Tabriz Health Services Management Research Center, School of Management & medical informatics, Tabriz University of Medical Sciences Tabriz Iran; ^3^ Community Based Participatory Research Center, Knowledge Utilization Research Center Tehran University of Medical Science Tehran Iran; ^4^ Family Medicine Memorial University of Newfoundland St. John's Canada

## Abstract

**Background:**

Review studies have reported on the low quality of study methodologies and poor reporting of knowledge translation (KT) interventional studies. This flaw cause the result of such studies to become misleading.

**Objectives:**

The present review is designed to evaluate the effect of methodological factors on the results of interventional studies that aimed to evaluate KT strategies at the policy level.

**Search Methods:**

Bibliographic databases and grey literature databases will be searched. The retrieved studies will be recorded in Covidence. After screening titles and abstracts, the full texts of selected studies will be assessed against the inclusion criteria. Disagreements will be resolved through discussion or by consultation with a third author.

**Selection Criteria:**

Primary studies are studies that aimed to estimate the efficacy of KT strategies to improve evidence‐informed policymaking. Study participants include policymakers and the intervention is a KT strategy. The main outcome is the desired changes in policy‐makers towards evidence‐informed decision‐making.

**Data Collection and Analysis:**

The main effect sizes will be expressed as standard mean difference and its variance for the main efficacy outcome of KT strategies in primary studies. Forest plot meta‐analysis will be used to synthesize the effect of each group of KT strategies. The contribution of ROB to the efficacy of KT interventions will be assessed via Meta‐epidemiology analysis. The overall estimate will be calculated using inverse‐variance random‐effects meta‐analysis with a 95% confidence interval for the estimate.

## 1 BACKGROUND

### 1.1 Summary

The low quality of study methodologies and poor reporting of Knowledge Translation (KT) interventional studies have been reported. This flaw cause the result of such studies to become misleading. The present review is designed to evaluate the effect of methodological factors on the studies that aimed to evaluate KT strategies at the policy level.

Bibliographic databases and grey literature databases will be searched. Eligible studies are studies that aimed to estimate the efficacy of KT strategies to improve evidence‐informed policymaking. Participants include policymakers and the intervention is a KT strategy. The main outcome is the desired changes in policy‐makers towards evidence‐informed decision‐making.

The main effect sizes will be expressed as standard mean difference and its variance for the main efficacy outcome of KT strategies in primary studies. Forest plot meta‐analysis will be used to synthesize the effect of each group of KT strategies. The contribution of ROB to the efficacy of KT interventions will be assessed via meta‐epidemiology analysis.

#### 1.1.1 The problem, condition, or issue

Public policy affects a large percentage of populations, and not using the best evidence in policymaking often leads to a waste of public resources. Therefore, to make better decisions, policymakers are required to be well‐informed about the latest evidence that relates to their policies (Oxman et al., 2009). KT strategies are defined as the collection of activities that are developed for using evidence in decision‐making more efficiently. KT is a dynamic and iterative process that includes a range of activities from producing research ideas to choosing ways to use evidence and evaluating intended changes in targeted audiences (CIHR, 2016; Lavis, 2003). A KT strategy can be an episode of giving interventional material(s) to individuals, audit and feedback, reminder, a modification in structure and services (EPOC, 2015), or other materials to make the desired change in at least one KT outcome in health policy (Armstrong, 2013; Sarkies, 2017). Relevant KT outcomes in health policy have been categorized into five groups (Edwards, 2019). The first category of KT outcomes is Instrumental changes that include changes in behaviours, practices, actions, decisions, plans and implemented policies. The second category which is called Conceptual is about changes in knowledge, awareness, attitudes, intentions, towards the desired behaviour. Capacity‐building is considered the third category of KT outcomes. It concerns changes to the skills and expertise of the targeted population. The fourth category is about changes to the number and quality of relationships and trust. This category is called Enduring connectivity. And the last category is changing in culture, attitudes and subjective norms towards knowledge exchange and research uptake.

However, despite implementing a wide range of KT strategies to inform policy‐makers about recent evidence, the efficacy of these strategies is not known (Sarkies, 2017). One reason for the lack of valid evidence about the efficacy of KT strategies is the low methodological quality of KT primary studies which had aimed to evaluate and compare KT strategies (LaRocca, 2012; Mitton, 2007; Sarkies, 2017; Scott, 2012). Many methodological limitations constrain the applicability of the results of KT comparative studies. Methodological limitations such as uncontrolled conditions, subjective outcomes, the complexity of KT interventions, inconsistency in the method of implementation of the strategy, hard‐to‐blind situations and the dependency of such strategies on contextual factors, usually push the validity of the results of such studies down and, consequently, several alternative explanations should be considered in the interpretation of the result of such studies (Boaz et al., 2011; Scott, 2012; Yost, 2015). Furthermore, methodological limitation raises challenges for comparing KT interventions in review studies (Boaz et al., 2011; Landry, 2001; Mitton, 2007; Oliver, 2014) and causes the lack of a solid base for choosing an appropriate KT strategy to be implemented in a specific condition (Armstrong, 2013; Bero, 1998; Mitton, 2007; Oliver, 2014; Tranfield, 2003). In addition to methodological constraints, contextual factors such as the level of the policy in which the KT strategies are implemented (Goor, 2017; LaRocca, 2012; WHO, 2018) can modify the observed efficacy of KT interventions. Although the effect of the contextual factors on the efficacy of KT interventions is not considered a limitation, the dependence of KT interventions on the contextual factors has often been over‐used to explain the observed heterogeneity in similar strategies (Boaz, 2011; Mitton, 2007).

There are solutions for estimating the effect of methodological quality characteristics of primary studies on the synthesized effect of interventions in review studies. One way is to perform the stratified meta‐analysis, considering the levels of methodological quality as strata (Higgins, 2011). Another approach is using the methodological quality of the individual studies as a study‐level covariate in meta‐regression analysis (Baker, 2009). Yet another possibility is to estimate the amount of contribution of the applied methodological techniques in primary studies to the synthesized efficacy via the Meta‐epidemiology approach (Bae, 2014). Meta‐epidemiology is a method of analysis that adopts the meta‐analysis approach to examine the impact of certain characteristics of primary studies on the synthesized measure and provides empirical evidence for hypothesized associations (Bae, 2014). This method tries to investigate the effect of the characteristics of primary studies on the estimated effect of interventions rather than the effect of the intervention itself (Bae, 2014; Murad, 2017; Sian, 2018). For example, it has been shown that independent of the estimated effect of a specific intervention in clinical trials, unblinded trials usually exaggerate the estimated effect of the targeted intervention, compared to blinded trials (Hróbjartsson, 2014). This example shows how methodological consideration can deviate from the result of a study and modify the estimated synthesized effect of an intervention in meta‐analysis.

Critical appraisal tools are used for measuring the level of methodological quality in different study designs. Since measuring the quality of the underlying research is not quite feasible, the concept of Risk of Bias (RoB) based on studies reports is used interchangeably (Higgins, 2011). Cochrane risk‐of‐bias tool for randomized trials (RoB 2) (Higgins, 2022) and the tool for assessing the risk of bias in non‐randomized studies of interventions (ROBINSI) (Sterne, 2016) are two validated and widely‐used tools to explore the level of risk of bias in randomized and non‐randomized studies, respectively. Combining RoB 2 and ROBINSI, there would be three groups of bias. First, biases that are exclusively related to randomized studies which are called Bias arising from the randomization process; second, biases for randomized and non‐randomized studies which include Bias due to missing outcome data, Bias in the measurement of the outcome and Bias in the selection of the reported result; and the third group are biases which are related to non‐randomized studies. They are categorized as Bias due to confounding, Bias in the selection of participants and Bias in the classification of intervention.

This study will use the meta‐epidemiological approach to investigate the effect of methodological quality on the observed efficacy of KT strategies. We consider the level of risk of bias (according to RoB 2 and ROBINSI) as a proxy for the methodological quality in primary studies. For each study, the association of the level of the risk of each bias and the overall risk of bias with the observed effect of KT strategies will be explored.

### 1.2 Description of the condition

In recent years, KT strategies have been developed for making use of evidence in decision‐making in different fields. KT is the process of making use of evidence in decision‐making that targets the sound implementation of research results. KT activities produce research ideas for defining actionable messages, choosing ways to use evidence and evaluating targeted changes in target audiences (Grimshaw, 2012; Straus, 2011). Despite applying many KT strategies to inform health policymakers about recent evidence, studies that ideally compare the efficacy of KT strategies aimed to arrive at the desired change in the policymaking process are uncommon and findings of many expensive studies do not provide proper evidence for practice (Sarkies, 2017). Review studies have reported on the low quality of study methodologies and poor reporting of KT primary studies (LaRocca, 2012; Mitton, 2007; Sarkies, 2017; Scott, 2012). This flaw may cause a significant distortion in the observed efficacy of such interventions (Scott, 2012; Yost, 2015) and consequently raises challenges for selecting an evidence‐based KT strategy in a specific condition (Bae, 2014; Bero, 1998; Mitton, 2007; Oliver, 2014). According to the Canadian Institutes of Health Research definition (CIHR, 2016), KT is a dynamic and iterative process that includes different activities and every piece of action that attempts to fill the ‘knowledge‐to‐action gap’ can be considered to be a KT intervention (Lavis, 2003). In fact, KT strategies are usually a type of complex intervention, consisting of several different components (Armstrong, 2013), and the synthesizing of the observed effect of a (so‐called) same intervention across different primary studies bears some extent of heterogeneity. In addition, contextual factors such as the environment in which the policy is made, the experience of the policymaker and the structure of the organizations implementing the interventions (Goor, 2017; LaRocca, 2012; WHO, 2018) can influence the observed effectiveness of KT interventions. This assortment might cause the effect of various methodological defects on the final observed effectiveness to be ignored by researchers in such studies (Boaz et al., 2011; Mitton, 2007). It has been shown that the dependence of the effect of KT intervention on local factors has been over‐used to explain the heterogeneity observed in studies results and causes the lack of a solid base for comparison of similar KT interventions in different primary studies (Landry, 2011; Oliver, 2014).

### 1.3 Description of the intervention

As mentioned above, variety in components of interventions and contextual factors might contribute to the effect of KT strategies. What's more, the methodological defects may introduce bias that limits the usefulness of studies. It takes place when imperfect methods of selection or flawed allocation methods are applied. Also, it might be aroused by the different performance of participants or study team's members respecting to study groups. Other methodological factors that can limit the usefulness of studies include the insufficient study sample size and the improper study design (Bae, 2014). Cochrane has suggested the concept of ‘risk of bias’ to measure whether a particular study is likely to be affected by the different biases (Higgins, 2011). Many tools and instruments have been proposed to assess the risk of bias in research studies. In the present review, the revised Cochrane risk‐of‐bias tool for randomized trials (RoB 2) and the tool for assessing the risk of bias in non‐randomized studies of interventions (ROBINSI) (Sterne, 2016) will be used to explore the level of risk of bias in randomized and non‐randomized studies, respectively. The Grading of Recommendations Assessment, Development, and Evaluation (GRADE) approach will be used to assess the quality of evidence for each estimate (Schünemann, 2013). Accordingly, the portion of the estimated effectiveness of KT strategies that can be attributed to the quality of the primary studies will be explored through the assessment of the relation between the risk of bias and the observed heterogeneity in the effectiveness of the studies. Factors that cause bias in primary studies (known as Meta‐confounders) (Bae, 2014) will be considered for further analysis.

### 1.4 How the intervention might work

To enhance the interpretability and the applicability of the review's results, it has been recommended that the heterogeneity due to the variety of intervention components and different contextual factors to be addressed in inclusion and exclusion criteria or be considered for subgroup analysis (Higgins, 2011). However, the heterogeneity in effect sizes that might be due to the different level of risk of bias should be considered separately (Bae, 2014; Schünemann, 2013). Cochrane Collaboration's recommends to present meta‐analyses stratified according to risk of bias or using meta‐regression to compare results from studies at high and low risk of bias to clarify the effect of risk of bias in the observed results. In the present review, a sort of analysis methods (Bae, 2014; Sian, 2018) would be applied for decomposition of the heterogeneity in KT interventional studies aimed to improve use of evidence in health policymaking. For each study, the methodological considerations that contribute to the risk of bias and overall judgement about risk of bias will be considered as intervention and their association with the observed effect of the apprised KT strategy will be explored through the between studies comparisons. It helps determine what portion of heterogeneity in studies results is attributable to the quality of studies.

### 1.5 Why it is important to do this review

Policymakers are responsible for choosing between alternatives. Their choices are more likely to improve results cost‐effectively when they are based on the best available evidence. In doing so, effective KT strategies should be implemented to inform policymakers of the evidence surrounding a particular policy or decision (Gavine, 2018; Oxman, 2009; Sarkies, 2017).

Despite the significant progress in developing KT strategies, in review studies that look for effective strategies for KT, inconsistency in the designs of studies and the difficulty of comparing the methodological quality of primary studies usually limit the usefulness of results. Although it is difficult to readily distinguish the contribution of different sources of variation to the effectiveness of KT strategies, their implications are completely different. Contextual factors (the effects of which are for the most part unknown) may act as effect modifiers and should be taken into account during the implementation phase. Mittonet and colleagues (2007) reviewed interventions to determine what KT strategies should be used in different health policy environments. They found that the amount of information available to assess a given KT strategy varied widely, and it is necessary to work more in this area to determine the application of KT strategies. They stated that there is insufficient evidence for conducting evidence‐based KT strategy in health policy decision‐making and emphasized primary research on KT interventions (Mitton, 2007). Boaz et al. (2011) conducted a review of reviews to evaluate the methods of implementation of evidence‐based findings into practice as opposed to other implementation strategies (nonevidence‐based). They identified very few systematic reviews looking exclusively and explicitly at KT studies. They criticized the assumption that all interventions (planned to improve clinical practice and health service) have been established based on high‐quality scientific evidence (Boaz et al., 2011).

The present review is designed to determine methodological factors that contribute to the heterogeneity of interventional studies aimed to evaluate KT strategies at the policy level. This approach will allow better estimation of the effectiveness of such strategies and will provide information to control bias for future studies. An array of methodological recommendations about how to evaluate KT strategies at the policy level in health‐related issues will be summarized.

The main audiences of this review are researchers who want to conduct primary interventional studies that aim to evaluate effectiveness of KT strategies for promoting evidence informed policy in health. In addition, the output of this review brings advantages for policymakers when they want to prioritize or select KT strategies. Summarized outlines of similar studies to the present review and what this review will add to, are presented in Table 1.

**Table 1:** Criteria and interpretation for Risk of Bias classification (Hinggs 2011)

Risk of bias	Interpretation	Criteria	Risk of bias
Low risk of bias	Unlikely to affect effect the magnitude or direction of results	Risk of all key domains of bias is low	Low risk of bias
Unclear risk of bias	It is not clear whether it will affect the magnitude or direction of results	One key domain of bias may change magnitude of results significantly	Unclear risk of bias
High risk of bias	Bias may change the magnitude or direction of results	At least one key domain of bias may change magnitude of results significantly or change the direction of the results	High risk of bias

## 2 OBJECTIVES

The main objective of this review is the assessment of the contribution of methodological quality to the efficacy of KT strategies in health policymaking. We will use RoB 2 and ROBINSI for measuring the risk of bias as the indication of methodological quality in randomized and non‐randomized studies. Then, the effect of the measured risk of bias on the observed effect sizes of each group of KT strategies will be estimated using meta‐epidemiology analysis. The secondary objective is to describe the applied methodological techniques over the types of KT strategies. We aimed to answer the following key questions in this study:

- To what extent does the level of the risk of bias in primary studies contribute to the estimated efficacy of KT strategies?
- To what extent, the observed heterogeneity between primary studies results can be attributed to the methodological quality in primary studies?
- What are the main methodological techniques that have been applied to reduce the risk of bias in KT studies in health policymaking?

**Figure 1:**
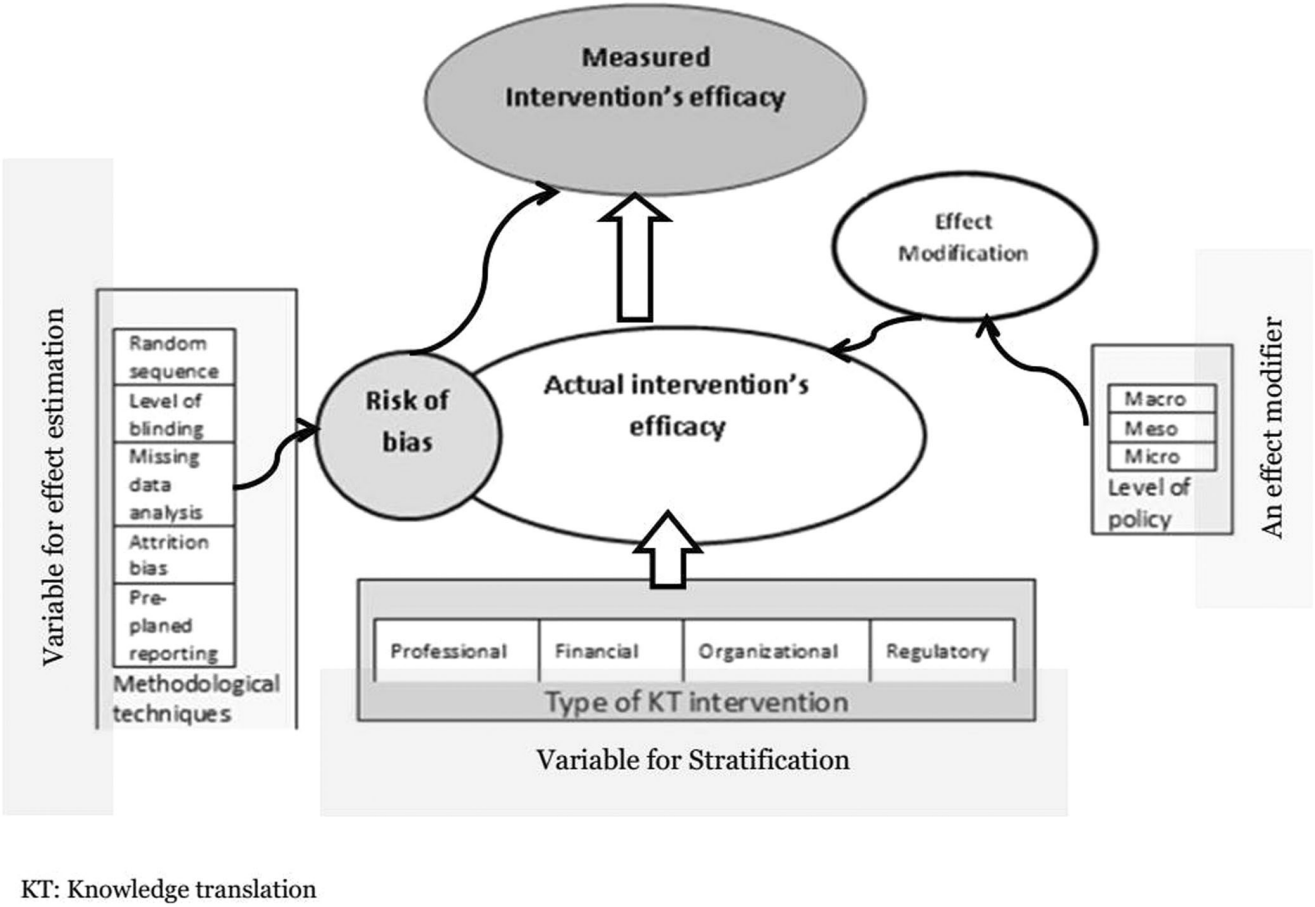
Determinants of measured (observed) efficacy of Knowledge translation interventions in health policy

The determinants (methodological quality and the type of KT strategy) and the level of policy(as an effect modifier) of the observed effects of KT strategies in primary studies and their presumed roles are depicted in Figure 1.

## 3 METHODS

### 3.1 Criteria for considering studies for this review

#### 3.1.1 Types of studies

This protocol was developed under the guidance of the Campbell Methods Coordination Group (Campbell collaboration, 2017).

Primary studies will include studies that aim to estimate the efficacy of KT strategies to improve evidence‐informed policymaking.

- Randomized trials: experimental studies in which participants are allocated to different study groups using random methods. It includes randomized controlled trials (RCTs), cluster‐randomized trials, cross‐over trials and pragmatic trials (EPOC, 2017).
- Non‐randomized trial: an experimental design in which participants are allocated to study groups using methods that are not random. It includes: non‐randomized control trials (NRT), controlled before‐after (CBA) studies and interrupted time series (ITS) and Repeated measures studies (EPOC, 2017).

Documents that are not in English will be accessed using translation software. If further clarity is required, the main study author(s) will be contacted.

#### 3.1.2 Types of participants

Study participants include individuals, groups, agencies, or regulatory bodies who make health decisions at local, national and international levels to improve population health outcomes.

#### 3.1.3 Types of interventions

The intervention is a KT strategy which can be an episode of giving interventional material(s) to individuals, audit and feedback, reminder, a modification in structure and services (EPOC, 2015). In addition, provider‐targeted strategies which include Educational material, Educational meeting, Educational outreach visit, Local opinion leader, Local consensus process, Peer review, Audit and feedback, Reminders and prompts, Tailored intervention, Patient‐mediated intervention and Multi‐faceted intervention (EPOC, 2015) will be considered for inclusion if they targeted policy‐makers as an independent group of participants. Other common KT strategies such as skill development, access to a knowledge broker, networking for information sharing and other resources and tools for evidence‐informed decision making (Armstrong, 2013; Sarkies, 2017) will be included if they were applied and investigated for evidence‐informed policy‐making.

#### 3.1.4 Types of outcome measures

The primary effect size is the standard mean difference and its variance. Wherever the main effect size in the selected studies is not reported by mean and variance, available data will be transformed via the Campbell collaboration effect size calculator (Polanin, 2016).

##### Primary outcomes

The primary outcome is the desired changes in policy‐makers towards evidence‐informed decision‐making that includes five categories (Edwards, 2019).

1. Instrumental: changes to behaviours, practices, actions, decisions, plans, policies
2. Conceptual: changes in knowledge, awareness, attitudes, intentions
3. Capacity‐building: changes to skills and expertise
4. Capacity‐building: changes to skills and expertise Enduring connectivity: changes in the number and quality of relationships and trust
5. Culture/attitudes/subjective norms towards knowledge exchange towards the research uptake.

Accordingly, some examples of KT outcomes that are being used in primary studies are as follows: Change in knowledge, attitude, practice; Presence of research results in policy; Increased importance for evidence‐based policy; Strengthen the relationship between researchers and policy‐makers; Production of action plans; Change in interests or ideas; Increased demands for KT products by policy‐makers; Increased access to research‐based evidence; Identification of gaps/conflicts/errors in policy and proposals.

If there is more than one outcome in a single included study, only the main measure will be considered. Whenever a study investigates a KT strategy with more than one primary outcome, the outcome that is more consistent with other studies will be chosen. Supposing that the two data extractors can not reach an agreement, the third reviewer would decide which outcome should be included.

###### Secondary outcomes

Funnel graph will be used to depict the distribution of ROB over the study effect sizes.

### 3.2 Search methods for identification of studies

The search strategy was developed based on Armstrong R, et al.'s study protocol (Armstrong, 2011) and Sarkies et al. review study (Sarkies, 2017). No date or language restrictions will be used. The search strategy for Medline via *ovid* is shown in Supporting Information Appendix 1. It will be adapted for the other databases.

#### 3.2.1 Electronic searches

- Bibliographic databases: MEDLINE(via Ovid), EMBASE, Cochrane central register of Controlled Trials, SCOPUS and Web of Science.
- Grey literature: Dissertation and theses: openSIGLE (opensigle.inist.fr), Proquest dissertations.

#### 3.2.2 Searching other resources

- *Other relevant sources*: previous reviews on the topic will be browsed through search in Cochrane Library, Campbell library, and PROSPERO to screen included studies against inclusion criteria. Other sources for finding relevant studies include:
  ∘ Database of Abstracts of Reviews of Effects https://www.crd.york.ac.uk/CRDWeb
  ∘ PDQ‐Evidence https://www.pdq-evidence.org/
  ∘ Knowledge translation methods and tools for public health www.nccmt.ca/knowledge-repositories/
  ∘ Health system evidence: https://www.healthsystemsevidence.org/
  ∘ EPPI‐Centre http://eppi.ioe.ac.uk
  ∘ The World Health Organization (WHO) http://www.who.int
  ∘ ESRC Evidence Network http://www.researchcatalogue.esrc.ac.uk/grants/H141251011/read

Targeted searching will be performed for articles that have been published in the *Journal of Implementation Science*, *Health Policy Journal*, *Health Policy and Planning*, *International Journal of Health Policy and Management*, *The International Journal of Health Planning and Management*, *BMC Public Health*, and *BMC Health Services Research* from 2006. In this stage, titles of all published articles in these journals since 2006, will be screened and those interventional studies with a group of policy‐makers as their participants will be included.

The reference list of included studies will be checked to find any other relevant study. There is no restriction on the date of publication and publication language. We also will search trial registers (ClinicalTrials.gov and the World Health Organization International Clinical Trials Registry Platform [ICTRP]) to identify registered (and completed) trials. The search strategy for Medline via *Ovid* is shown in Supporting Information Appendix 1. It will be adapted for the other databases.

### 3.3 Data collection and analysis

Publications, that are suspected to be repeated reports of one set of data will be explored, and the most relevant publication will be used for final analysis. If there is more than one measure per outcome in the included studies, only one measure per study will be used for each quantitative data synthesis.

Where designs other than individually allocated design (such as stratified randomization, cluster randomization or individual matching) are included, they will be assessed to determine if the correlation was considered in sample size calculation or data analysis. In the case that the design was not properly accounted, the correction will be made through an appropriate statistical method to make a direct estimate of the required effect measure. A corrected measure of effect and its confidence interval will be considered for the quantitative data synthesis.

If there is more than one eligible comparison from one study, for data synthesis, combining groups to create a single pairwise comparison will be considered. If it is not sensible to combine intervention groups, correlated comparisons will be included, accounting for the correlation. Supposing that this method is not possible, other solutions such as splitting the sample size of a common group into two or more groups with a smaller sample size will be considered. When all these methods do not work appropriately, one comparison with the highest sample size will be selected and others will be excluded.

‘Summary of findings’ table will be provided to depict a summary of information across KT strategies. The amount of evidence, the estimated effect on up‐taking evidence by policymakers, the quality of the body of evidence, characteristics of the setting, and the type of policy that was concerned more, will be considered to be included in the table.

#### 3.3.1 Selection of studies

The retrieved studies will be recorded in Covidence systematic review software (Available at www.covidence.org). After removing duplicates references, the titles and abstracts will be screened by two independent reviewers to determine which will meet the inclusion criteria. In doing so, the first 10% of the articles will be assessed to provide sufficient calibration for this stage. The full text of selected studies will be obtained, and the two reviewers will explore them to determine their eligibility for inclusion. The full‐text studies that do not meet the inclusion criteria will be excluded from the review at this stage, and reasons for exclusion will be provided in the table of ‘Characteristics of excluded studies’. The screening and selection process will be reported in the final report and outlined in a PRISMA flow diagram (Moher et al., 2009). This process will be done using the predefined inclusion and exclusion criteria. Disagreements will be resolved through discussion or by consultation with a third author. Publications that are suspected to be repeated reports of one set of data will be explored and the most relevant publication will be used for final analysis.

#### 3.3.2 Data extraction and management

Data extraction will include the following characteristics:

- Citations: authors, publication year, journal and language
- Types of KT intervention: the episode of giving interventional material(s) to individuals, audit and feedback, reminder or a modification in structure and services.
- The main KT outcome per study (if it is not the same as the study primary outcome)
- Study participants: age, sex and other factors.
- Risk of Bias: as described in the section of Quality Appraisal
- Methodological techniques to reduce risk of bias: (sequence generation, allocation concealment, blinding, incomplete outcome data analysis, others)

Supposing that the two data extractors could not reach an agreement upon the main outcome of a study, the third reviewer would decide which outcome should be included. The data extraction form will be examined by the selection of 10 included studies before it will be used for all included studies. The *κ* index will be calculated to evaluate inter‐reviewer agreement (*κ* > 0.7 is a good agreement). The discordant items will be reviewed, and the tool will be revised if required. Data extraction will be performed by two reviewers independently (Supporting Information Appendix 3). Disagreement between reviewers will be resolved through discussion. If the disagreement remains, the third author will make the final decision.

#### 3.3.3 Assessment of risk of bias in included studies

According to the mentioned tools, a specific bias will be classified as ‘low risk’ if its risk is as low as it could not change the magnitude or distort the direction of the study results. If it is not clear whether that bias affected the magnitude or the direction of results, it will be considered ‘unclear’ and finally, a ‘high risk’ bias is a bias that may significantly change the magnification/direction of study results. To explore bias from selective reporting, the outcomes in the protocols and published reports will be compared. If the protocol is not available, the study will be downgraded for reporting bias. Supporting information, supplemented with reviewer comments, with a quote extracted from the study report and a justification for decisions (high, low, or unclear) for each bias will be provided according to the Cochrane Handbook (Higgins, 2011) (Table 1). Table 2 is the designed template of the data gathering form for RoB in RCTs and non‐RCTs.

**Table 2:** Template for the results of the risk of bias assessment in RCTs and nRCTs

	Type of bias	Study #1	Study #2	Study #3
RCTs	Bias arising from the randomization process	H□ l□ U□	H□ l□ U□	H□ l□ U□
		Supporting information	Supporting information	Supporting information
		Justification for decision	Justification for decision	Justification for decision
RCTs and nRCTs	Bias due to deviations from intended interventions	H□ l□ U□	H□ l□ U□	H□ l□ U□
		Supporting information	Supporting information	Supporting information
		Justification for decision	Justification for decision	Justification for decision
	Bias due to missing outcome data	H□ l□ U□	H□ l□ U□	H□ l□ U□
		Supporting information	Supporting information	Supporting information
		Justification for decision	Justification for decision	Justification for decision
	Bias in measurement of the outcome	H□ l□ U□	H□ l□ U□	H□ l□ U□
		Supporting information	Supporting information	Supporting information
		Justification for decision	Justification for decision	Justification for decision
	Bias in selection of the reported result	H□ l□ U□	H□ l□ U□	H□ l□ U□
		Supporting information	Supporting information	Supporting information
		Justification for decision	Justification for decision	Justification for decision
nRCTs	Bias due to confounding	H□ l□ U□	H□ l□ U□	H□ l□ U□
		Supporting information	Supporting information	Supporting information
		Justification for decision	Justification for decision	Justification for decision
	Bias in selection of participants	H□ l□ U□	H□ l□ U□	H□ l□ U□
		Supporting information	Supporting information	Supporting information
		Justification for decision	Justification for decision	Justification for decision
	Bias in classification of intervention	H□ l□ U□	H□ l□ U□	H□ l□ U□
		Supporting information	Supporting information	Supporting information
		Justification for decision	Justification for decision	Justification for decision

#### 3.3.4 Measures of treatment effect

The main effect sizes will be expressed as the standard mean difference and its variance for the main efficacy outcome of KT strategies in primary studies. Wherever the effect size was not presented by means and variance, available data will be transformed via the Campbell collaboration effect size calculator (Polanin, 2016).

#### 3.3.5 Unit of analysis issues

Standard mean difference.

#### 3.3.6 Dealing with missing data

When data for studies outcomes were not stated the authors of the papers will be contacted.

#### 3.3.7 Assessment of heterogeneity

Heterogeneity across different KT strategies will be assessed using Cochran's *Q* and the *I* ^2^ statistic.

#### 3.3.8 Assessment of reporting biases

To explore bias from selective reporting the outcomes in the protocol and published report will be compared. If the protocol is not available, the study will be downgraded for reporting bias.

#### 3.3.9 Data synthesis

Forest plot meta‐analysis will be used to synthesize the effect of each group of KT strategies. The contribution of ROB to the efficacy of KT interventions will be assessed via meta‐epidemiology analysis that includes synthesizing the standardized mean differences (SMD) of KT outcomes across KT primary studies, considering ROB conditions as the determinant factor (Bae, 2014; Sian, 2018). To perform meta‐epidemiological analysis, all studies will be categorized as low risk of bias and high or unknown risk of bias (according to RoB 2 and ROBINSI). A forest plot meta‐epidemiological analysis, presenting the effect of the level of risk of bias will be drawn. For any type of KT intervention, at least one study with low ROB and one study with high ROB are required.

The overall estimate will be calculated using an inverse–variance random‐effects meta‐analysis, with a 95% confidence interval for the estimate.

Furthermore, the association of the score of ROB with the observed heterogeneity will be assessed through meta‐regression. Meta‐regression refers to the regression‐based method of analysis that evaluates the association between study‐level converts and the combined effect (Higgins, 2011). To perform further analysis, the meta‐regression will be used and the type of KT intervention and the level of policy is used as a covariate in the model.

Finally, a funnel graph will be used to depict the distribution of ROB over the primary studies' effect sizes.

#### 3.3.10 Subgroup analysis and investigation of heterogeneity

Different types of KT strategies shall be considered for subgroup analysis.

##### Adequacy of data

In a primary search, we found many studies that potentially meet the inclusion criteria (169 clinical trials were retrieved via PubMed, which had at least one keyword about knowledge translation in their title or abstracts). Sarkies and colleagues found 19 primary studies had evaluated different types of research implementation strategies for promoting evidence‐informed policy and management decisions in healthcare. Their review included published studies up to February 2016 (Sarkies, 2017). According to these findings, we believe we would have enough independent studies to perform a quantitative synthesis.

The contribution of the estimated ROB to the observed heterogeneity will be assessed through meta‐regression.

#### 3.3.11 Sensitivity analysis

If data are not available sensitivity analysis via the base case scenario will be applied.

#### 3.3.12 Summary of findings and assessment of the certainty of the evidence

A descriptive analysis of the findings from the selected studies structured around the type of methods, the type of the study's intervention, type of study's outcomes and the level of policy (macro, meso and micro) will be provided. For each study, the type of methodological considerations and overall judgement about the methodological quality and their association with the combined effect of KT strategies will be considered for description.

## CONTRIBUTIONS OF AUTHORS

AA will retrieve data from included studies and perform the statistical analysis and draft the protocol and the final report. LD will retrieve data from included studies and make contribution to the policymaking concept. RM and BY will contribute to elaborate knowledge translation concept and method. SA will make contribution to the methodology of the review and drafting the protocol and final report. All authors will check all parts of the review in term of content and methods.

- Content: AA, LD, RM, BY
- Systematic review methods: AA, SA
- Statistical analysis: AA, SA
- Information retrieval:AA, LD

## DECLARATIONS OF INTEREST

All authors declare no conflict of interest.

## SOURCES OF SUPPORT

**Internal sources**

• Tehran University of Medical Sciences, Iran

This study is funded by Tehran University of Medical Sciences, No: 95‐02‐159‐32240

**External sources**

• New Source of support, Other.

## Supporting information

Supporting information.
