# Supplementary material for: PROTOCOL: Systematic review of methods to reduce risk of bias in knowledge translation interventional studies in health‐related issues
Source: Campbell Syst Rev. 2022 Apr 12;18(2):e1236. doi: 10.1002/cl2.1236 (PMC9005927; doi:10.1002/cl2.1236)
Supplement: Supplementary file 1 — Supporting information. [file CL2-18-e1236-s001.docx]

# Appendices

## 1 Search strategy (Medline via ovid)

| 1. (knowledge adj2 (application or broke$ or creation or diffus$ or disseminat$ or exchang$ or implement$ or management or mobili$ or translat$ or transfer$ or uptak$ or utili$)).ti,ab. |  |
| --- | --- |
| 2. (evidence$ adj2 (exchang$ or translat$ or transfer$ or diffus$ or disseminat$ or exchang$ or implement$ or management or mobil$ or uptak$ or utili$)).ti,ab. |  |
| 3. (KT adj2 (application or broke$ or diffus$ or disseminat$ or decision$ or exchang$ or implement$ or intervent$ or mobili$ or plan$ or policy or policies or strateg$ or translat$ or transfer$ or uptak$ or utili$)).ti,ab. |  |
| 4. (research$ adj2 (diffus$ or disseminat$ or exchang$ or transfer$ or translation$ or application or implement$ or mobil$ or transfer$ or uptak$ or utili$)).ti,ab. |  |
| 5. ("research findings into action" or "research to action" or "research into action" or "evidence to action" or "evidence to practice" or "evidence into practice").ti,ab. |  |
| 6. (("research utilis$" or "research utiliz$") and ("decision mak$" or decisionmak$ or decision-mak$ or "policy mak$" or "policymak$" or "policy decision$" or "health$ polic$" or practice or action$1)).ti,ab. |  |
| 7. Diffusion of Innovation/ or (diffusion adj2 innovation).ti,ab. |  |
| 8. (("systematic review$" or "knowledge synthes$") adj5 ("decision mak$" or "policy mak$" or "policy decision?" or "health polic$")).ti,ab. |  |
| 9. (("systematic review$" or "knowledge synthes$") adj2 (application or implement$ or utili?ation or utilize? orutilise? orutili? ing)).ti,ab. |  |
| 10. Research utili?ation.ti,ab. |  |
| 11. ((evidence base$ or evidence inform$) adj5 (decision$ or plan$ or policy or policies or practice or action$)).ti,ab. |  |
| 12. 1 or 2 or 3 or 4 or 5 or 6 or 7 or 8 or 9 or 10 or 11 |  |
| 13. Public Health/ |  |
| 14. Public Health Administration/ |  |
| 15. Public Health Practice/ |  |
| 16. Community Health Services/ or Community health planning/ |  |
| 17. community health$.ti,ab. |  |
| 18. Health Promotion/ |  |
| 19. health promotion?.ti,ab. |  |
| 20. exp public policy/ |  |
| 21. (health$ adj2 (policy$ or policies)).ti,ab. |  |
| 22. exp health planning/ and (decision? or decision-mak$ or policy or policies).ti,ab,hw. |  |
| 23. "health care (non mesh)"/ or *population characteristics/ or public health administration/ |  |
| 24. exp health policy/ |  |
| 25. 13 or 14 or 15 or 16 or 17 or 18 or 19 or 20 or 21 or 22 or 23 or 24 |  |
| 26. (randomized controlled trial or controlled clinical trial or clinical trial).pt. |  |
| 27. random$.ti,ab. |  |
| 28. controlled.ti. |  |
| 29. (control$ adj (clinical or group$ or trial$ or study or studies or design$ or method$)).ti,ab. |  |
| 30. control groups/ |  |
| 31. single-blind method/ or double-blind method/ |  |
| 32. (intervention? ormultiintervention? or multi-intervention? or postintervention? or post-intervention? or preintervention? or preintervention?).ti,ab. |  |
| 33. ("pre test$" or pretest$ or posttest$ or "post test$").ti,ab. |  |
| 34. (control$ adj2 (before or after)).ti,ab. |  |
| 35. ("quasi-experiment$" or quasi experiment$ or "quasi random$" or quasirandom$ or "quasi control$" or quasicontrol$ or ((quasi$ or experimental) adj3 (method$ or study or studies or trial or design$))).ti,ab. |  |
| 36. "time series".ti,ab,hw. |  |
| 37. 'multicenter study'.pt. |  |
| 38. (multicent$ adj2 (design? or study or studies or trial?)).ti,ab. |  |
| 39. follow-up studies/ or follow up/ |  |
| 40. (('follow up' or follow-up) adj2 (design or study or studies)).ti,ab. |  |
| 41. cross-over studies/ or crossover procedure/ |  |
| 42. ((crossover or cross-over) adj2 (design or study or studies or trial)).ti,ab. |  |
| 43. Comparative study.pt. or comparative study/ |  |
| 44. (comparative adj2 (study or studies)).ti,ab. |  |
| 45. intervention studies/ or intervention study/ |  |
| 46. program evaluation/ |  |
| 47. evaluation studies.pt. |  |
| 48. ('evaluation study' or 'evaluation studies').ti,ab. |  |
| 49. ((Process or program$) adj3 (effect$ or evaluat$)).ti,ab. |  |
| 50. follow-up assessment.ti,ab. |  |
| 51. 26 or 27 or 28 or 29 or 30 or 31 or 32 or 33 or 34 or 35 or 36 or 37 or 38 or 39 or 40 or 41 or 42 or 43 or 44 or 45 or 46 or 47 or 48 or 49 or 50 |  |
| 52. (audit or self-audit).ti,ab,hw. |  |
| 53. (booklet$ or brochure? or pamphlet? or paper-based or 'printed material?').ti,ab. |  |
| 54. "barrier? and facilitator?".ti,ab. |  |
| 55. decision making/ or decision mak$.ti,ab,hw. |  |
| 56. ((change? or changing or improv$ or effect$ or influenc$ or alter$ or adapt$ or amend$ or modify$ or adjust$ or transform$) adj2 (policy or policies or process$ or practic$ or provider? or activit$)).ti,ab. |  |
| 57. ((knowledge or evidence or quality or research or practice) adj2 gap?).ti,ab. |  |
| 58. (education$ adj3 (continuing or group? or outreach or plan$ or practitioner? or program? or staff? or team?)).ti,ab,hw. |  |
| 59. ("evidence based" adj3 (algorithm? or evaluat$ or guideline? or healthcare or implement$ or improv$ or intervention$ or management or pathway? or plan? or practic$ or program? or quality)).ti,ab. |  |
| 60. (feedback not (feedback adj loop$)).ti,hw. |  |
| 61. Guideline Adherence/ |  |
| 62. (guideline? adj3 (adher$ or enforc$ or influenc$ or implement$ or impact$ or introduc$ or uptake or follow)).ti,ab. |  |
| 63. (incentiv$ adj2 (economic or employee? or financ$ or insurer? or insurance or market$ or monetar$ or pay$ or plan? or practitioner? or program$ or provider? or reimburs$ or salary or salarie? or staff or team$ or value-based)).ti,ab. |  |
| 64. (collaborat$ or 'cross-profession$' or intraprofession$ or intra-profession$ or interprofession$ or inter-profession$ or (skill adj2 mix$) or teambase? or team-based or inter disciplin$ or multidisciplin$ or multi disciplin$ or multiprofession$).ti,ab,hw. |  |
| 65. ((knowledge adj2 (transfer$ or translation or shar$ or exchan$)) or KT).ti,ab. |  |
| 66. ((knowledge or evidence or practice) adj2 (gap? or barrier?)).ti,ab. |  |
| 67. ((knowledge or evidence) adj2 synthesis).ti,ab. |  |
| 68. 'opinion leader?'.ti,ab. |  |
| 69. (outreach adj2 (communit$ or plan? or program? or visit?)).ti,ab. |  |
| 70. ((policy or policies) adj2 (chang$ or effect? or impact? or influenc$)).ti,ab. |  |
| 71. (quality adj2 (assurance or improvement? or initiativ$ or plan$ or program$ or review or audit)).ti,ab. |  |
| 72. (QI adj (inititative? or intervention? or program$ or plan$ or audit)).ti,ab. |  |
| 73. computers, handheld/ or handheld?.ti,ab. or (PDA or 'personal data assistant?' or blackberr$).ti,ab. |  |
| 74. telephon$.ti,ab,hw. or (tele-health or tele-medicine or e-health).ti,ab. |  |
| 75. internet.ti,ab,hw. or (intranet or LAN or WAN or blog$ or (computer$ adj2 network$) or online$ or web$ or wiki).ti,ab. |  |
| 76. Social marketing/ or 'social marketing'.ti,ab. |  |
| 77. 'virtual communit$'.ti,ab. |  |
| 78. ((change? or changing or improv$ or effect$ or influenc$) adj2 (policy or policies or practic$ or provider?)).ti,ab. |  |
| 79. ('performance based' or value-based).ti,ab. |  |
| 80. 52 or 53 or 54 or 55 or 56 or 57 or 58 or 59 or 60 or 61 or 62 or 63 or 64 or 65 or 66 or 67 or 68 or 69 or 70 or 71 or 72 or 73 or 74 or 75 or 76 or 77 or 78 or 79 |  |
| 81. 12 and 25 and 51 and 80 |  |

## 2 Study' Codebook

[Enter text here]

| **General Information** | | | | | | | | | | | | | | | | | | | | | | | |
| --- | --- | --- | --- | --- | --- | --- | --- | --- | --- | --- | --- | --- | --- | --- | --- | --- | --- | --- | --- | --- | --- | --- | --- |
| Review title or ID | | | | | | | | | |  | | | | | | | | | | | | | |
| Study ID *(surname of first author and year first full report of study was published)* | | | | | | | | | |  | | | | | | | | | | | | | |
| Report ID | | | | | | | | | |  | | | | | | | | | | | | | |
| Report ID of other reports of this study | | | | | | | | | |  | | | | | | | | | | | | | |
| Notes | | | | | | | | | | | | | | | | | | | | | | | |
| Date form completed *(dd/mm/yyyy)* | | | | | | |  | | | | | | | | | | | | | | | | |
| Name/ID of person extracting data | | | | | | |  | | | | | | | | | | | | | | | | |
| Study author contact details | | | | | | |  | | | | | | | | | | | | | | | | |
| Notes: | | | | | | | | | | | | | | | | | | | | | | | |
| **Study eligibility** | | | | | | | | | | | | | | | | | | | | | | | |
| Study Characteristics | | Eligibility criteria  *(Insert inclusion criteria for each characteristic as defined in the Protocol)* | | | | | | | | | | | Eligibility criteria met? | | | | | | | Location in text or source *(pg & ¶/fig/table/other)* | | | |
|  |  |  |  |  |  |  |  |  |  |  |  |  | Yes | | No | | Unclear | | |  |  |  |  |
| Type of study | | Randomised Controlled Trial | | | | | | | | | | |  | |  | |  | | |  | | | |
|  |  | non-randomised Controlled Trial | | | | | | | | | | |  | |  | |  | | |  | | | |
| Participants | | Groups, agencies or regulatory bodies who make health decisions in local, national and international level to improve populations health outcomes. | | | | | | | | | | |  | |  | |  | | |  | | | |
| Types of intervention | | KT strategy which can be an episode of giving interventional material(s) to individuals, audit & feedback, reminder, a modification in structure and services  and other materials in order to make a desired change in at least one KT outcome in health policy | | | | | | | | | | |  | |  | |  | | |  | | | |
| Types of comparison | | No Intervention or routine practice | | | | | | | | | | |  | |  | |  | | |  | | | |
| Types of outcome measures | | The ultimate outcomes of KT strategies are research impact measures in policy making that includes 5 categories | | | | | | | | | | | Instrumental  Conceptual  Capacity-building: changes to skills and expertise  Enduring connectivity  Culture/attitudes/ subjective norms towards knowledge exchange and towards research uptake. | | | | | | |  | | | |
| INCLUDE | | | | | | | | | | | EXCLUDE | | | | | | | | | | | | |
| Reason for exclusion | |  | | | | | | | | | | | | | | | | | | | | | |
| Notes:  **DO NOT PROCEED IF STUDY EXCLUDED FROM REVIEW** | | | | | | | | | | | | | | | | | | | | | | | |
| **Characteristics of included studies** | | | | | | | | | | | | | | | | | | | | | | | |
| **Methods** | | | | | | | | | | | | | | | | | | | | | | | |
|  | | | **Descriptions as stated in report/paper** | | | | | | | | | | | | | | | | **Location in text or source***(pg & ¶/fig/table/other)* | | | | |
| **Aim of study** | | |  | | | | | | | | | | | | | | | |  | | | | |
| **Design** | | | Randomised Controlled Trial  randomized controlled trials, cluster randomized trials, cross over trials and pragmatic trials  Non-randomised Controlled Trial  non- randomized control trials (NRT), controlled before-after (CBA) studies and interrupted time series (ITS) and Repeated measures studies | | | | | | | | | | | | | | | |  | | | | |
| **Unit of allocation** | | |  | | | | | | | | | | | | | | | |  | | | | |
| **Start date** | | |  | | | | | | | | | | | | | | | |  | | | | |
| **End date** | | |  | | | | | | | | | | | | | | | |  | | | | |
| **Duration of participation** | | |  | | | | | | | | | | | | | | | |  | | | | |
| **Notes:** | | | | | | | | | | | | | | | | | | | | | | | |
| **Participants** | | | | | | | | | | | | | | | | | | | | | | | |
|  | | | | Description | | | | | | | | | | | | | | | Location in text | | | | |
| Population description | | | |  | | | | | | | | | | | | | | |  | | | | |
| Setting | | | |  | | | | | | | | | | | | | | |  | | | | |
| Inclusion criteria | | | |  | | | | | | | | | | | | | | |  | | | | |
| Exclusion criteria | | | |  | | | | | | | | | | | | | | |  | | | | |
| Method of recruitment of participants | | | |  | | | | | | | | | | | | | | |  | | | | |
| Total no. participants | | | |  | | | | | | | | | | | | | | |  | | | | |
| Level of policy | | | | Macro  Meso  Micro | | | | | | | | | | | | | | |  | | | | |
| Notes: | | | | | | | | | | | | | | | | | | | | | | | |
| **Intervention groups** | | | | | | | | | | | | | | | | | | | | | | | |
| **Intervention Group 1** | | | | | | | | | | | | | | | | | | | | | | | |
|  | | | | | | Description as stated in report/paper | | | | | | | | | | | | | | | | Location in text or source | |
| KT Strategy name | | | | | |  | | | | | | | | | | | | | | | |  | |
| KT Strategy type | | | | | | episode of giving interventional material(s) to individuals, audit & feedback, reminder, a modification in structure and services others : | | | | | | | | | | | | | | | |  | |
| No. participants to group | | | | | |  | | | | | | | | | | | | | | | |  | |
| Description | | | | | |  | | | | | | | | | | | | | | | |  | |
| Duration of intervention period | | | | | |  | | | | | | | | | | | | | | | |  | |
| Timing | | | | | |  | | | | | | | | | | | | | | | |  | |
| Delivery | | | | | |  | | | | | | | | | | | | | | | |  | |
| Providers | | | | | |  | | | | | | | | | | | | | | | |  | |
| Notes: | | | | | | | | | | | | | | | | | | | | | | | |
| **Outcomes**  *Copy and paste table for each outcome.* | | | | | | | | | | | | | | | | | | | | | | | |
| **Outcome 1** | | | | | | | | | | | | | | | | | | | | | | | |
|  | | | | | Description as stated in report/paper | | | | | | | | | | | | | | Location in text or source *(pg & ¶/fig/table/other)* | | | | |
| Outcome name | | | | |  | | | | | | | | | | | | | |  | | | | |
| Outcome type | | | | | Instrumental  Conceptual  Capacity-building: changes to skills and expertise  Enduring connectivity  Culture/attitudes/ subjective norms towards knowledge exchange and towards research uptake. | | | | | | | | | | | | | |  | | | | |
| Time points measured | | | | |  | | | | | | | | | | | | | |  | | | | |
| Time points reported | | | | |  | | | | | | | | | | | | | |  | | | | |
| Outcome definition | | | | |  | | | | | | | | | | | | | |  | | | | |
| Person measuring/ reporting | | | | |  | | | | | | | | | | | | | |  | | | | |
| Unit of measurement | | | | |  | | | | | | | | | | | | | |  | | | | |
| Scales: upper and lower limits | | | | |  | | | | | | | | | | | | | |  | | | | |
| Is outcome/tool validated? | | | | | Yes         No          Unclear | | | | | | |  | | | | | | |  | | | | |
| Notes: | | | | | | | | | | | | | | | | | | | | | | | |
| **Data and analysis** | | | | | | | | | | | | | | | | | | | | | | | |
|  | | | | | Description as stated in report/paper | | | | | | | | | | | | | | | | Location in text or source | | |
| Comparison | | | | |  | | | | | | | | | | | | | | | |  | | |
| Outcome | | | | |  | | | | | | | | | | | | | | | |  | | |
| Subgroup | | | | |  | | | | | | | | | | | | | | | |  | | |
| Time point (specify from start or end of intervention) | | | | |  | | | | | | | | | | | | | | | |  | | |
| Results | Intervention | | | | | | | | | | Comparison | | | | | | | | | |  | | |
|  | Mean | | SD *(or other variance, specify)* | | | | | No. participants | | | Mean | | | SD *(or other variance, specify)* | | | | No. participants | | |  |  |  |
|  |  | |  | | | | |  | | |  | | |  | | | |  | | |  |  |  |
| Notes: | | | | | | | | | | | | | | | | | | | | | | | |
| **Dichotomous outcome** | | | | | Intervention | | | | | | | Comparison | | | | | | | | |  | | |
|  |  |  |  |  | No. with event | | | | Total in group | | | No. with event | | | | Total in group | | | | |  |  |  |
|  |  |  |  |  |  | | | |  | | |  | | | |  | | | | |  |  |  |
| Any other results reported | | | | |  | | | | | | | | | | | | | | | |  | | |
| Unit of analysis | | | | |  | | | | | | | | | | | | | | | |  | | |
| Notes: | | | | | | | | | | | | | | | | | | | | | | | |
| **Other information** | | | | | | | | | | | | | | | | | | | | | | | |
|  | | | | | | **Description as stated in report/paper** | | | | | | | | | | | | | | | | | **Location in text or source** |
| **Applied Methodological techniques** | | | | | | random sequence generation allocation concealment blinding of participants and  blinding of personnel;  blinding of outcome assessors  incomplete outcome data  selective outcome reporting  baseline comparability of participant characteristics between groups  consistency in intervention delivery and  using validated data gathering tools | | | | | | | | | | | | | | | | |  |
| **Key conclusions of study authors** | | | | | |  | | | | | | | | | | | | | | | | |  |
| **Notes:** | | | | | | | | | | | | | | | | | | | | | | | |
